# Evaluation of the Surface Hardness and Roughness of a Resin-Modified Glass Ionomer Cement Containing Bacterial Cellulose Nanocrystals

**DOI:** 10.1155/2021/8231473

**Published:** 2021-12-11

**Authors:** Maryam Saadat, Marzieh Moradian, Babak Mirshekari

**Affiliations:** ^1^Oral and Dental Disease Research Center, Department of Operative Dentistry, School of Dentistry, Shiraz University of Medical Sciences, Shiraz, Iran; ^2^Student Research Committee, School of Dentistry, Shiraz University of Medical Sciences, Shiraz, Iran

## Abstract

The purpose of this study was to evaluate the performance of a resin-modified glass ionomer cement (RMGIC) to which bacterial cellulose nanocrystals (BCNs) were added. BCNs were incorporated into the RMGIC powder in ratios of 0.3%, 0.5%, and 1% (w/w). One control and three experimental groups were enrolled in the study: unmodified RMGIC (control), 0.3% (w/w) BCN-modified RMGIC, 0.5% (w/w) BCN-modified RMGIC, and 1% (w/w) BCN-modified RMGIC. The surface hardness and surface roughness were the parameters assessed. The materials were characterized by scanning electron microscopy (SEM). The data were analyzed using the one-way ANOVA and Kruskal–Wallis tests for surface hardness and roughness, respectively. The addition of BCN resulted in the improvement of surface roughness in all the specimens compared with the control material. The RMGIC modified by 1% (w/w) BCN showed the lowest surface roughness (decreased by 52%) among all tested groups. However, BCN had a negative effect on the surface hardness of RMGIC. The group with 0.3% (w/w) BCN had the least decrease in microhardness (13%). According to the results, the RMGIC group modified by 1% (w/w) BCN had a smoother surface than the other groups. The surface microhardness of the RMGIC decreased after BCNs were added to it.

## 1. Introduction

The glass ionomer cement (GIC) is an attractive dental restorative material to replace tooth tissue loss from caries lesion [1]. GICs have advantageous properties including low thermal expansion coefficient, biocompatibility, adhesion to the tooth structure, antimicrobial action, and anticariogenic capability [2–5]. However, they also have unfavorable physical and mechanical properties such as poor polishability, sensitivity to dehydration, and moisture contamination during the early stages of setting and the formation of cracks and gaps [6].

Resin-modified GICs (RMGICs) were first introduced as bases and liners. However, they were further modified to overcome their early moisture sensitivity and low mechanical properties so that they could be used as direct restorative materials [7–9]. Similar to GIC materials, RMGICs contain not only the conventional acid-base reaction but also a resin monomer polymerization which can be activated either chemically or by light [10]. These materials have the same composition as conventional GICs with the addition of polyacrylic acid. However, the polymerizable resin monomer in RMGIC is commonly 2-hydroxyethyl methacrylate (HEMA) [10]. HEMA in RMGICs leads to a greater water uptake and the swelling of the resin matrix due to its hydrophilic composition [11] which might in turn deteriorate the mechanical properties of the cement [12].

Recently, different nanoparticles and bioactive nanoceramics have been incorporated into the GIC in order to overcome the mentioned limitations [13–15]. The integration of nanoparticles such as nanotitanium dioxide (TiO2), nanosilicon dioxide (SiO2), nanozirconium dioxide (ZrO2), nanochitosan, and nanohydroxyapatite into restorative materials has significantly increased their mechanical properties [16–21] by enhancing particle distribution, surface area, and surface energy. Among these nanostructured materials, reinforcement with cellulose nanocrystals (CNCs) is a technical application in dentistry and medicine. CNCs have several advantages such as low cost, low density, easy fabrication process, and high specific mechanical properties compared with other nanostructured materials [22]. Apart from plants, certain bacteria are also known to produce cellulose in a relatively pure form. Bacterial cellulose is unique because of its high crystallinity, high water-holding capacity, and excellent thermomechanical properties [23].

The surface microhardness and roughness of dental restorative materials are important. The surface roughness of dental materials is affected by both intrinsic and extrinsic factors. Compared with smooth surfaces, rough surfaces cause more plaque accumulation, and the material is more easily worn. An increase in the surface roughness of restorative materials is a preparatory factor for bacterial colonization as well as a risk factor for gingival diseases which could develop in the future [24]. Moradian et al. [25] described the use of bacterial cellulose nanocrystals (BCNs) in enhancing the mechanical properties of RMGIC. The authors observed that the addition of an appropriate proportion of BCNs to RMGICs increased their compressive and diametral tensile strengths. However, no previous study has investigated the microhardness and surface roughness values of RMGICs modified with BCNs. Therefore, the aim of the present study was to analyze the effect of incorporating different proportions of BCNs into RMGIC on their mechanical properties. The null hypothesis of the current in vitro study was that the addition of BCNs to the RMGIC would not affect their surface roughness (SR) and surface hardness (SH).

## 2. Materials and Methods

### 2.1. Grouping of Specimens

The factor under study was the incorporation of different concentrations of BCNs (0.3%, 0.5%, and 1% by weight) into RMGICs (Fuji II LC, GC Corporation, Tokyo, Japan). The specimens were divided into four different experimental groups: unmodified RMGIC (control), 0.3% (w/w) BCN-modified RMGIC, 0.5% (w/w) BCN-modified RMGIC, and 1% (w/w) BCN-modified RMGIC. The evaluated parameters included the surface roughness (*n* = 8/group) and surface hardness (*n* = 8/group). This study was conducted after the approval of the Research Ethics Committee of Shiraz University of Medical Sciences (IR.SUMS.DENTAL.REC.1399.062).

### 2.2. Specimen Preparation

The BCN powder (Nano Novin Polymer Co., Gorgan, Golestan, Iran) at three different concentrations (0.3%, 0.5%, and 1% in weight) was added to the RMGIC powder (containing 95% fluoroaluminosilicate glass (amorphous) and 5% polyacrylic acid) and then mixed with the RMGIC liquid (20–30% distilled water, 20–30% polyacrylic acid, and 30–35% HEMA). The bacterial cellulose was extracted from *Gluconacetobacter* genus. A precision scale accurate to 0.0001 g (GR-3000, A & D CL Toshiba, Tokyo, Japan) was used to determine the weights of the RMGIC and BCN powders. After weighing the materials, BCNs were manually added to the RMGIC powder at different concentrations (0.3%, 0.5%, and 1% by weight) as previously mentioned [25]. The materials were mixed following the manufacturer's instructions for powder/liquid ratio (3/2 g: 1 g). The specimens were prepared at room temperature. In brief, the RMGIC with or without BCN was placed in Teflon molds, inserted in a single increment, and pressed between polyester strips under a glass slide with a static load. The specimens were light-cured for 20 seconds through the polyester strip using a light-emitting diode (GT 1200, BluLEX, Monitex, Taiwan) with the light intensity of 1200 mW/cm^2^ at the wavelength range of 420–490 nm. After removing the specimens, they were light-cured from the other side to ensure that they were completely cured. Next, the specimens were stored for 7 days at 37°C and 100% humidity before performing the tests.

### 2.3. Surface Roughness Test

Thirty-two disc-shaped specimens (with the diameter of 7 mm and height of 2 mm) were prepared (*n* = 8/group) and wet-polished using 400-, 800-, and 1200-grit silicon carbide papers to obtain uniform surfaces. Then, they were cleaned by ultrasound for 10 min (TESA Rugosurf 20, Switzerland). The SR of the specimens was analyzed using a surface profilometer (TESA Rugosurf 20, Switzerland) in five different positions. The needle moved at a constant speed of 0.5 mm/s with a tracing length of 4 mm and a cutoff value of 0.8 mm.

### 2.4. Surface Microhardness Test

Similar to the SR test, 32 disc-shaped specimens (with the diameter of 7 mm and height of 2 mm) were prepared (*n* = 8/group), wet-polished, and cleaned by ultrasound. The Vickers microhardness test was carried out in a digital microhardness tester (SCTMC®, MHV-1000Z, China) using a diamond indenter with 300 gf loads and a dwell time of 15 s for five indentations across each specimen. The Vickers hardness number (VHN) was calculated as the mean of the five readouts taken.

### 2.5. Surface Characterization by Scanning Electron Microscopy (SEM)

In order to determine the impact of BCNs on the structure of RMGICs, the specimens were submitted to SEM analysis (TESCAN Vega III, Czech Republic) (*n* = 1/group). Briefly, the RMGIC specimens were prepared as described above (with or without BCNs), left to dry for 24 h, sputtered with a thin gold layer, and analyzed at 1000 × magnification at a working distance of 10 mm at 20 kV.

### 2.6. Statistical Analysis

The data were analyzed using the Shapiro–Wilk and Kolmogorov–Smirnov normality tests (*p* ≤ 0.05). The microhardness of the groups was compared using the analysis of variance (ANOVA) test followed by Tukey's test, whereas the Kruskal–Wallis test and Dunn's post hoc test were used to assess the statistical differences in roughness between the control group (without BCN treatment) and the BCN groups at different concentrations. All the analyses were performed using the SPSS software (version 18, SPSS Inc., Chicago, USA).

## 3. Results

**Figure 1:**
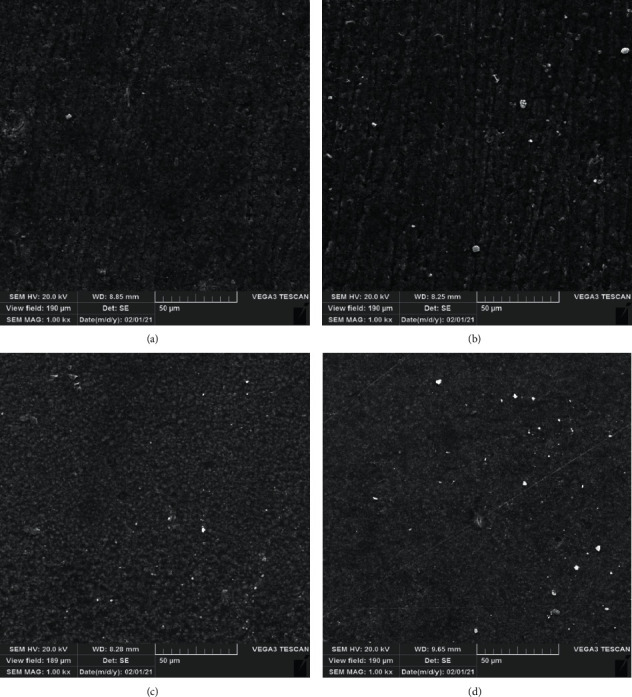
Scanning electron microscopy micrographs of the specimen surfaces with 1000 × magnification: (a) the control group, (b) the 0.3% (w/w) BCNs-modified RMGIC group, (c) the 0.5% (w/w) BCNs-modified RMGIC group, and (d) the 0.3% (w/w) BCNs-modified RMGIC group.

The one-way ANOVA test demonstrated that the unmodified RMGIC (control) group had the highest SH mean values compared to the other experimental groups (*p* ≤ 0.05). In addition, the intergroup analysis revealed that the 0.3% (w/w) BCN group had higher SH values than the 0.5% (w/w) (*p*=0.047) and 1% (w/w) BCN (*p*=0.001) groups. There were no significant differences between the two latter groups regarding SH. In the present study, the intergroup analysis further revealed that SR significantly decreased in the specimens with the addition of 0.3%, 0.5%, and 1% (w/w) BCN compared with the control group (*p* ≤ 0.05) (Table 1).  Figure 1 shows the SEM characterization of the impact of BCN on the RMGIC structure. The surface morphology of the RMGIC modified by BCN exhibited a higher degree of integrity and a smoother surface compared with the control group.

## 4. Discussion

The present study evaluated the mechanical properties (SR and SH) of a RMGIC modified by the addition of BCNs. Cellulose is the most available organic compound on the planet and has become a natural resource for fabricating organic elements (such as cellulose microfibers and nanocrystals) for reinforcements. These particles have several advantages such as renewable resources, low cost, low density, high mechanical properties, nonabrasive characteristics, and easy processing [22]. According to Silva et al. [26], the different concentrations of cellulose nanocrystals (CNCs) used to produce the specimens were due to the relative size and properties of the CNC particles. The CNC concentrations of over 1% led to the aggregation of the nanoparticles and the failure of mechanical properties. The advantage of CNC is that very low concentrations of it are needed for reinforcement due to the large specific area of the nanoparticles.

Surface roughness is created by numerous physical processes. The average roughness (Ra) is the most frequently used parameter to describe surface roughness and is evaluated with a profilometer [27]. Surface roughness affects gingival inflammation, bacterial adhesion, stain resistance, and dental plaque accumulation of restoration [28]. The results of this study revealed substantial improvements in SR of RMGIC reinforced with BCN nanofillers compared with that of unmodified RMGIC materials. These findings can be explained by the fact that the nanosized BCNs affected the distribution between the particles and the matrix and the interfacial bonding between the particles. These findings are in accordance with previous studies suggesting that the particle size is an important factor affecting the surface roughness of dental materials [29,30] and that using nanosized particles may lead to a favorable surface roughness for dental materials. Therefore, it can be concluded that the addition of BCN to RMGIC will positively affect its surface roughness. In line with this hypothesis, the SEM analysis in the current research showed that the addition of BCNs altered the surface structure of RMGICs as shown in Figure 1. The SEM photographs showed that the surfaces with higher BCNs had low porosities and voids compared to the control surface.

Surface hardness shows the localized surface resistance of a material to indentation and is associated with the underlying material matrix. The experimental surface microhardness of the BCN-modified RMGIC was reduced compared with that of the unmodified RMGIC regardless of the BCN concentration used. This showed that the inclusion of BCN nanofillers did not enhance the surface hardness of the RMGICs which is in line with the work of Silva et al. [26]. Higher BCN concentrations may weaken the bulk of the cement because this nanocrystal is probably not chemically attacked by the acid and is not able to react in acid–basic reaction to take part in the setting of RMGICs. Moreover, the weakening of the cement bulk decreases the ability to resist indentation and therefore reduces hardness [31].

In the current research, the decreased SH in all the BCN-modified groups compared with that of the unmodified RMGIC could be explained by the presence of a larger number of glass particles at the surface of the control group. Also, it might be explained by heterogeneous phases in the BCN-modified cement, but energy-dispersive X-ray spectroscopy (EDS) is needed to identify the elemental compositions of materials, particles, and to better explain the relationship between VHN value and RMGICs.

On the other hand, small-sized BCNs have a greater surface area than glass particles. As a result, higher concentrations of BCNs would lead to an insufficient amount of polyacrylic polymer for reaction. This weakens the internal crosslinking and reduces SH [32]. In addition, SH of the RMGICs may be affected by other factors such as the size and morphology of the fillers [33] as well as the insufficient dispersion of BCN and glass particles.

It is important to highlight that the results of SR in this study were better than the results of SH. Several studies have demonstrated the good mechanical properties of GICs reinforced with cellulose nanocrystals [25, 34]. This shows the potential of cellulose nanocrystals to improve GICs as a whole which can replace dental amalgam in the future.

The future of RMGICs modified with BCNs depends on further studies on their color stability and clinical tests for a complete understanding of the properties and characteristics of these materials particularly in the oral environment.

## 5. Conclusions

Considering the limitations of this study, it can be concluded that the surface roughness and microhardness of RMGICs were significantly affected by the incorporation of BCN particles. The RMGIC modified by 1% BCN had the smoothest surface. On the other hand, the addition of BCNs to the RMGIC significantly decreased its microhardness compared with that of the control group.

## Figures and Tables

**Table 1:** Mean and standard deviation of microhardness and surface roughness of the studied groups.

	Microhardness (VHN)	Roughness (*μ*m)
Test groups (*n* = 8)	Mean ± SD	Mean ± SD
Control (unmodified RMGIC)	41.60 ± 3.43^a^	0.96 ± 0.33^a^
0.3% (w/w) BCN-modified RMGIC	36.19 ± 2.38^b^	0.59 ± 0.09^b^
0.5% (w/w) BCN-modified RMGIC	31.71 ± 4.48^c^	0.60 ± 0.13^b^
1% (w/w) BCN-modified RMGIC	29.35 ± 2.16^c^	0.46 ± 0.13^b^

The columns with the same lower-case character are not significantly different from each other (*p* > 0.05).

## Data Availability

The data used to support the findings of this study are available from the corresponding author upon request.
